# Expression of transcript factors SALL4 and OCT4 in a subset of non-small cell lung carcinomas (NSCLC)

**DOI:** 10.1186/s40247-014-0010-7

**Published:** 2014-10-02

**Authors:** Erika Rodriguez, Li Chen, Ming-Hui Ao, Susan Geddes, Ed Gabrielson, Frederic Askin, Hui Zhang, Qing Kay Li

**Affiliations:** Department of Pathology, The Johns Hopkins Medical Institutes, 4940 Eastern Ave., Baltimore, 21287 MD USA

**Keywords:** Transcription factor SALL4 and OCT4, Immunohistochemistry (IHC), Non-small cell lung cancer (NSCLC), Metastatic NSCLC, Lung cancer biopsy and tissue microarray (TMA)

## Abstract

**Background:**

SALL4 and OCT4 are transcription factors and play essential roles in stem cell development and oncogenesis. However, the expression of these transcription factors has not been well studied in lung cancers. In this study, we evaluated the expression of SALL4 and OCT4 in non-small cell lung carcinomas (NSCLC) by immunochemistry. NSCLC tissue microarrays (TMAs) were constructed with a total of 77 primary lung adenocarcinomas (ADCs) and 90 primary lung squamous cell carcinomas (SqCCs). A mouse monoclonal anti-human SALL4 (1:400 dilution) and a polyclonal anti-human OCT4 (1:200 dilution) antibodies were used. Nuclear staining of SALL4 and OCT4 was scored semi-quantitatively using a three tiered scale. The expressions of SALL4 and OCT4 were correlated with the tumor differentiation, pathological stage, and patients' clinical information.

**Results:**

In primary ADCs, the stronger expression of SALL4 and OCT4 was 7.8% and 9.1%, respectively. The stronger expression of SALL4 was inversely correlated with tumor differentiations. In primary SqCCs, the stronger expressions of SALL4 and OCT4 were 16.7% and 0%, respectively. The expression of SALL4 is correlated with the expression of OCT4, but inversely correlated with the tumor stage in SqCCs.

**Conclusions:**

We found that both SALL4 and OCT4 were differentially expressed in a subset of primary ADC and SqCC. Our finding suggest that different stem cell markers may be expressed and/or play differential role in different subtypes of NSCLC. The potential role of SALL4 and OCT4 needs to be further investigated in NSCLC.

**Electronic supplementary material:**

The online version of this article (doi:10.1186/s40247-014-0010-7) contains supplementary material, which is available to authorized users.

## Background

Lung cancer is one of the most common cancers worldwide and in the United States [1]. It is the most common cause of cancer-related death in both sexes [1]. Morphologically, lung cancer can be divided in two major histological groups, non small cell (NSCLC) and small cell carcinoma (SCLC). NSCLC is the most common type and represents approximately 80% of all lung cancers [2],[3]. In NSCLCs, 70% of patients present with advanced or metastatic disease at the diagnosis [1]-[3]. Recent molecular studies of NSCLC find that mutations of the *EGFR* gene are present in 10-20% of lung adenocarcinomas (ADC) and are associated with successful treatment with EGFR inhibitors [4],[5], whereas *KRAS* mutations are present in 20-30% of ADC and associated with non-response to EGFR inhibitors [4]-[6]. Furthermore, the identification of *EML4*-*ALK* gene translocation in 3% of ADC leads to targeted therapy with the ALK inhibitor crizotinib [7]. NSCLC is, however, a heterogeneous group of neoplasms, with different morphologic subtypes, epigenetic and molecular abnormalities, making targeted therapy and treatment a challenge [4],[6],[8]-[10]. Therefore, the separation of diverse lung cancer phenotypes and gentotypes, and the identification of novel markers have become critically important for making therapeutic decisions [4],[6]-[11].

In recent years, the stem cell theory has become an important paradigm in cancer development and progression. This theory proposes that tumor cells are organized in a hierarchical manner, where cancer stem cells (CSC) represent an undifferentiated cell population, analogous to embryonic stem cells [12]-[15]. CSCs have the ability to self renew and give rise to a pool of undifferentiated CSC, as well as more differentiated progeny cancer cells, that form the bulk of the tumor mass [12]-[15]. CSCs have been identified in hematologic malignancies as well as in solid tumors such as melanoma, breast, brain, prostate, pancreas and lung carcinomas [12]-[15]. In the signaling network of CSC, several molecules such as Sal-like protein 4 (SALL4), octamer-binding transcription factor 4 (OCT4), Nanog and Klf4, have been identified as important factors in maintaining the stem cell's self-renewal and dividing ability [16]-[18]. Based on the finding that SALL4 regulates the activation and expression of OCT4, Klf4 and other proteins [16]-[18], SALL4 has been identified as one of the master transcription factors in the molecular network which maintains the pluripotency of stem cells [17],[18]. Recently, a few studies have provided the evidence that SALL4 also plays an important role in oncogenesis [19]-[23]. For example, the expression of SALL4 has been found in a subset of leukemia/lymphomas [19], malignant rhabdoid tumors [20], germ cell tumors [21], liver, and stomach carcinomas [22]. The expression of SALL4 has also been correlated with different stages of colorectal [23] and breast cancers [24].

In lung cancer, the expression of CSC markers OCT4 and Nanog has been reported [25], however, the expression of SALL4 in NSCLC is still not well known. In this study, we investigated the expression of SALL4 and OCT4 using NSCLC tumor tissue microarrays (TMAs) and immunohistochemistry; and correlated their expressions with histological features of tumors and clinical information of patients. The purpose of our study is to examine the potential involvement of these CSC markers in NSCLC.

## Methods

### Lung cancer tissue microarray (TMA) construction

The lung carcinoma tissue microarray (0.6 mm in diameter, 3-4 cores per case) was constructed using surgical resected specimens retrieved from the department of pathology archives at The Johns Hopkins Hospitals over a period of ten years (from 1999 to 2009). Tumor tissues were fixed in 10% buffered formalin and embedded in paraffin. A hematoxylin and eosin (H&E) stained section of the paraffin-embedded tumor sample was reviewed by the pathologist (QKL) to select the tumor area for TMA construction. The World Health Organization criteria were used to determine histological subtypes of lung NSCLC [2],[3]; and AJCC (American Joint Committee on Cancer) 7^th^ edition [26] was used to determine the pathological stage of the tumor at the time of diagnosis. A total of 77 cases of primary lung ADCs and 90 cases of primary lung SqCCs were included.

All tumor cases were annotated with available clinical information in a manner that protected patient identity. The use of human tumor tissue was approved by the Johns Hopkins Institutional Review Board.

### Immunohistochemistry (IHC)

IHC was performed on TMAs using the Dako autostainer from the clinical immunohistochemistry laboratory at the Johns Hopkins Hospitals. Sections of TMAs were cut at 4 microns thickness and deparaffinized prior to incubation with primary antibodies. Heat antigen retrieval at 70°C for 40 minutes was also used to enhance signal detection. Primary antibodies were diluted according to standard protocols and manufacturer suggestions. A mouse monoclonal antibody against human SALL4 (clone 6e3, 1:400 dilution, SIGMA, St Louis, MO), and a mouse polyclonal antibody against human OCT4 (clone C-2, 1:200 dilution, ABCAM, Cambridge, MA) were used. IHC of nuclei was scored based on the intensity and percentage of stains using a semi-quantitatively three tiered scale: 0 = negative, 1 = focally and weakly positive (<20%), and 2 = positive (>20%). The IHC stains on TMAs were scored by ER, MHA and QKL. If there was a disagreement, the consensus was reached by discussion of the case among reviewers. Appropriate positive and negative controls were included in the IHC assay.

### Statistical analysis

Characteristics of clinical information and pathological variables among different subsets of tumors were correlated with the expression of SALL4 and OCT4. The Student's t-test and Fisher's exact test were used. All tests were two-sided with the p-values less than 0.05 to be considered statistically significant.

## Results and discussion

### Clinical information

In primary lung ADC cases, the patients' median age was 63 years (range from 45 to 86 years). The male: female ratio was: 1:0.97. Among tumors, 40 cases were pT1, 31 cases were pT2, and 6 cases were pT3/4 tumors. The mean tumor size was 3.07°Cm, ranging from 0.5 to 9.0°Cm. The subtypes of ADCs were as follows: mixed (44 cases), acinar (19 cases), mucinous (6 cases), true papillary (4 cases), solid (3 cases), and non-mucinous ADC with lepidic pattern (formerly bronchioloalveolar adenocarcinoma, 1 case). In SqCC cases, the patients' median age was 64 years (range from 40 to 86 years). The male: female ratio was: 1:0.60. Among tumors, 25 cases were pT1, 28 cases were pT2, 26 cases were pT3 and 11 cases were pT4 tumors. The mean tumor size was 4.32°Cm, ranging from 0.20 to 13.5°Cm. Clinical information was summarized in Table 1. We did not find any significant differences of patients' age and gender between ADC and SqCC (p > 0.05, p = 0.9631 and p = 0.1314, respectively). The average tumor size of SqCC is 4.32°Cm, and it was significantly larger than the average size of 3.07 cm in ADCs (p < 0.001, p = 0.0003).

**Table 1:** **Clinical information of patients**

Characteristics	Adenocarcinomas(n = 77)	Squamous cell carcinomas(n = 90)	P values
Gender (cases (%))			0.1314
Male	39 (50.6%)	56 (62.2%)	
Female	38 (49.4%)	34 (37.8%)	
Age (years)			0.9631
Average ± SD	62.97 ± 13.62	63.06 ± 11.49	
Range	45-86	40-86	
Tumor size (cm)			0.0003
Average ± SD	3.07 ± 1.81	4.32 ± 2.73	
Range	0.5- 9.0	0.2-13.5	
Pathological stage (case (%))			N/A
pT1	40 (51.9%)	25 (27.8%)	
pT2	31 (40.3%)	28 (31.1%)	
pT3	4 (5.2%)	26 (28.9%)	
pT4	2 (2.6%)	11 (12.2%)	

SD: standard deviation. N/A: not applicable.

### Expression of SALL4 and OCT4 in normal tissue and primary NSCLC

In our study, 112 normal tissues were included as controls in TMAs. The expression of SALL4 and OCT4 were summarized in Table 2. Immunoreactivities of SALL4 and OCT4 were negative in 75% (n = 84) and 79% (n = 88) cases, respectively. In SALL4, 9 cases (8%) were 2+ scores, and most of these cases were found in gastrointestinal (GI) epithelium. The weak and focal immunoreactivity were found in GI, germinal center cells of lymphoid tissue, kidney tubular cells, basal cells of bronchial epithelium, prostate, skin, and placental trophoblasts. Similarly, in OCT4, the most immunoreactivity was found in the skin, GI epithelium, prostate, lung and few other tissues.

**Table 2:** **Expressions of SALL4 and OCT4 in normal tissues**

Tissue	SALL4 expression			OCT4 expression		
	Negative(0)	Score(1+)	Score(2+)	Negative(0)	Score(1+)	Score(2+)
Lymphoid tissue (n = 22)	19	3	0	22	0	0
Kidney (n = 11)	10	1	0	9	2	0
Smooth muscle (n = 4)	4	0	0	4	0	0
Brain (n = 5)	5	0	0	5	0	0
Fibroconnective tissue (n = 9)	9	0	0	9	0	0
Lung (n = 10)	9	1	0	7	3	0
Skin (n = 2)	1	1	0	0	0	2
Placenta (n = 6)	3	3	0	5	1	0
Pancreas acini (n = 5)	5	0	0	5	0	0
Salivary glands (n = 2)	2	0	0	2	0	0
Prostate (n = 7)	3	3	1	4	3	0
GI tract (n = 29)	14	7	8	16	13	0
**Total (n = 112, 100%)**	**84 (75%)**	**19** **(17%)**	**9** **(8%)**	**88** **(79%)**	**22** **(20%)**	**2** **(1%)**

**Figure 1:**
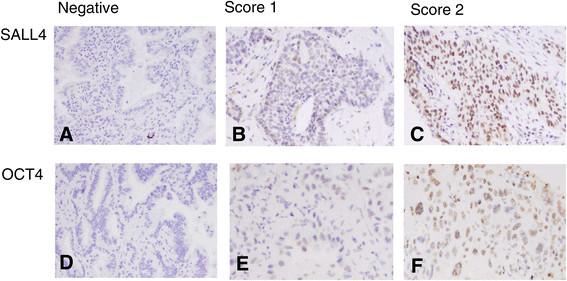
**SALL4 and OCT4 expressions in NSCLC. A**-**C**, immunostains of SALL4 in NSCLC. **D**-**F**, immunostains of OCT4 in NSCLC. Score 0 = negative, score 1 = focally and weakly positive (<20%), and score 2 = positive (>20%).

The expression of SALL4 and OCT4 in primary lung ADC and SqCC was summarized in Table 3 and Figure 1. In 77 ADCs, the expression of SALL4 was negative in 66.2%, weakly positive in 26.0% and positive in 7.8% cases. In 90 SqCCs, SALL4 was negative in 62.2%, weakly positive in 21.1% and positive in 16.7% of cases. The stronger expression of SALL4 was found more frequent in SqCC than that in ADC. The expression of OCT4 in these two groups was as follows: in ADC, OCT4 was negative in 62.3%, weakly positive in 27.3% and positive in 9.1% cases, whereas, the expression of OCT4 in SqCCs was negative in 74.4%, weakly positive in 25.6% and positive in 0% cases. The stronger expression of OCT4 was found more frequent in ADC than that in SqCC.

**Table 3:** **IHC stains of SALL4 and OCT4 in primary lung ADC and SqCC**

NSCLC	SALL4 expression			OCT4 expression			
	Negative	Score	Score	Negative	Score	Score	N/A
	(0)	(1+)	(2+)	(0)	(1+)	(2+)	
ADC (n = 77)	51 (66.2%)	20 (26.0%)	6 (7.8%)	48 (62.3%)	21 (27.3%)	7 (9.1%)	1* (1.3%)
SqCC (n = 90)	56 (62.2%)	19 (21.1%)	15 (16.7%)	67 (74.4%)	23 (25.6%)	0 (0%)	

N/A: not applicable.

*One case did not have tumor tissue on the TMA.

We further examined SALL4 and OCT4 expression in different subtypes of ADC (Tables 4 and 5). The stronger expression of SALL4 was found in several subtypes of ADC, whereas, OCT4 expression was found predominately in the mixed subtype of ADCs.

**Table 4:** **Expression of SALL4 in subtypes of lung adenocarcinomas**

Subtype of adenocarcinoma	SALL4 expression		
	Negative	Score	Score
	(0)	(1+)	(2+)
**Mixed** **(n =** **44)**	30 (68.2%)	13 (29.6%)	1 (2.3%)
**Acinar** **(n =** **19)**	13 (68.4%)	3 (15.8%)	3 (15.8%)
**Mucinous** **(n =** **6)**	4 (66.7%)	1 (16.7%)	1 (16.7%)
**True papillary** **(n =** **4)**	3 (75.0%)	1 (25.0%)	0 (0.0%)
**Solid** **(n = 3)**	0 (0.00%)	2 (66.7%)	1 (33.3%)
**Lepidic (n = 1)**	1 (100.0%)	0 (0.0%)	0 (0.0%)
**Total (n = 77)**	51 (66.2%)	20 (26.0%)	6 (7.8%)

**Table 5:** **Expression of OCT4 in subtypes of lung adenocarcinomas**

Subtype of adenocarcinoma	OCT4 expression			
	Negative	Score	Score	N/A
	(0)	(1+)	(2+)	
**Mixed (n = 44)**	23 (52.3%)	14 (31.8%)	6 (13.6%)	1 (2.3%)*
**Acinar (n = 19)**	15 (77.0%)	4 (21.1%)	0 (0.0%)	
**Mucinous (n = 6)**	5 (83.3%)	1 (16. 7%)	0 (0.0%)	
**True papillary (n = 4)**	2 (50.0%)	2 (50.0%)	0 (0.0%)	
**Solid (n = 3)**	3 (100.0%)	0 (0.0%)	0 (0.0%)	
**Lepidic (n = 1)**	0 (0.00)	0 (0.0%)	1 (100.0%)	
**Total (n = 77)**	48 (62.3%)	21 (27. 3%)	7 (9.1%)	1 (1.3%)

N/A: not applicable.

*One case did not have tumor tissue on the TMA.

Both SALL 4 and OCT4 play important roles in embryonic development and oncogenesis. Human *SALL4* gene is the homolog of the Drosophila gene *sal*-*like 4*[27],[28] and located on the long (q) arm of chromosome 20 at position 13.2 [28]. The functional role of SALL4 has been suggested as the essential transcript factor in the maintenance of stem cell pluripotency and CSC proliferation [16]-[18]. In addition to play an important role in human embryonic development, mutation of *SALL4* gene has been related to several congenital and developmental abnormalities, such as DRRS (Duane-Radial Ray Syndrome) and acro-renal-ocular syndrome [29]. In animal studies, the constitutive expression of *SALL4* in transgenic mice has been linked to the development of acute myeloid leukemia and lymphocytic leukemia [30]. In human, the overexpression of *SALL4* has been reported in hematologic malignancies such as acute myelocytic and lymphocytic leukemia [19]; and in solid tumors such as malignant rhabdoid tumor [20], germ cell tumor [21], liver and stomach [22], colorectal [23] and breast cancers [24]. Furthermore, SALL4 has also been detected in Wilms tumor, but not in nephrogenic rests, this observation suggests that SALL4 could be used as a protein marker in the distinction of these two entities [31]. Similarly, OCT4 is also an important regulator of stem cell differentiation and plays an essential role in embryogenesis [25],[32]. OCT4 expression has been reported in different types of cancers and involved in cancer progression [32].

In lung cancers, the expression of OCT4 and Nanog has been reported [25],[32], however, the expression of SALL4 in NSCLC has not been well studied. Our study by using lung cancer TMA and immunohistochemistry demonstrated that both SALL4 and OCT4 are expressed in a subset of NSCLC. In primary ADCs, the stronger expression of SALL4 and OCT4 was 7.8% and 9.1%. In primary SqCCs, the stronger expressions of SALL4 and OCT4 were 16.7% and 0%, respectively. The stronger expression of SALL4 is more frequently detected in SqCCs than in ADCs. In contrast, the stronger expression of OCT4 is more frequently detected in lung ADCs.

### Expressions of SALL4 and OCT4 among differently differentiated tumors

**Figure 2:**
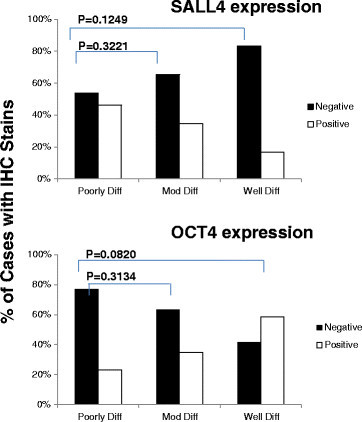
**SALL4 and OCT4 expressions in differently differentiated primary lung adenocarcinomas (ADCs).** In 77 ADCs, 12 cases were well differentiated, 52 cases were moderately differentiated and 13 cases were poorly differentiated. The expressions of SALL4 and OCT4 showed different patterns.

**Figure 3:**
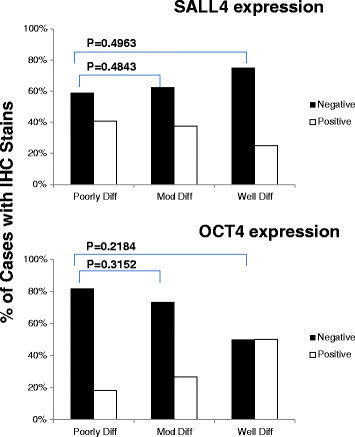
**SALL4 and OCT4 expressions in differently differentiated primary lung squamous cell carcinomas (SqCCs).** In 90 SqCCs, 4 cases were well differentiated, 64 cases were moderately differentiated and 22 cases were poorly differentiated. The expressions of SALL4 and OCT4 showed different patterns.

We also examined and correlated expressions of SALL4 and OCT4 among differently differentiated tumors. Data were summarized in Figures 2 and 3. In 77 ADCs, 12 cases were well differentiated, 52 cases were moderately differentiated and 13 cases were poorly differentiated. The expressions of SALL4 and OCT4 showed different patterns (Figure 2). The stronger SALL4 expression was found more frequently in poorly differentiated ADCs, and it was inversely correlated with the tumor differentiation. In contrast, the stronger OCT4 expression was found more frequently in well differentiated ADCs. The inversed relationship of SALL4 and OCT4 expression and tumor differentiation was found. However, the statistical analysis did not found significant differences between groups.

In 90 SqCCs, 4 cases were well differentiated, 64 cases were moderately differentiated and 22 cases were poorly differentiated. The expressions of SALL4 and OCT4 showed different patterns (Figure 3). The stronger SALL4 expression was found in both poorly and moderately differentiated SqCC. But, this pattern was not found in OCT4 expressions. The OCT4 expression was more frequent in well differentiated SqCCs. However, the statistical analysis did not found significant differences between groups.

Recent studies of the molecular mechanism of SALL4 in stem cells have shown that SALL4 activates transcriptional factor OCT4, interacts with Nanog, and forms a protein-protein complex in SALL4/ Oct4/Nanog signaling pathway [18]. More recently, the detection of SALL4 in germ cell tumors [21] suggests the clinical utility of this protein as a biomarker for diagnosing primary and metastatic germ cell tumors in a variety of anatomic sites such as brain, testes, ovary and mediastinum. In our study, we observed that SALL4 was more frequently expressed in poorly differentiated tumors, whereas, OCT4 was more frequently expressed in well differentiated tumors. Our data suggested that SALL4 and OCT4 may play a differential role in the process of tumor differentiation.

### Correlation of SALL4 and OCT4 expression between tumor stage and patients' survival time

**Figure 4:**
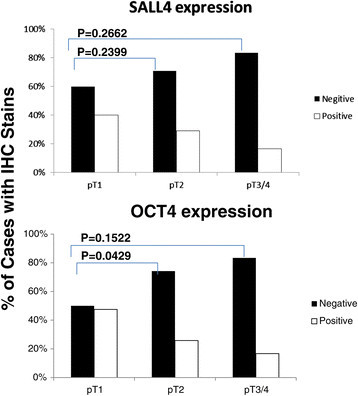
**SALL4 and OCT4 expressions in different stages of primary lung adenocarcinomas (ADCs).** In 77 ADCs, 40 cases were pT1, 31 cases were pT2, and 6 cases were pT3/4 tumors. The stronger expressions of SALL4 and OCT4 were found more frequently in pT1 ADCs.

**Figure 5:**
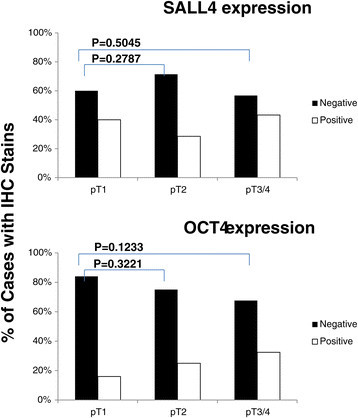
**SALL4 and OCT4 expressions in different stages of primary lung squamous cell carcinomas (SqCCs).** In 90 SqCCs, 25 cases were pT1, 28 cases were pT2, and 37 cases were pT3/4 tumors. The expressions of SALL4 and OCT4 showed different patterns. The stronger SALL4 expression was found in all different stages of SqCCs. The stronger OCT4 expression was more frequently in pT3/4 SqCCs.

The expressions of SALL4 and OCT4 among different pathological stages of tumors were summarized in Figures 4 and 5. In 77 ADCs, 40 cases were pT1, 31 cases were pT2, and 6 cases were pT3/4 tumors. The stronger expressions of SALL4 and OCT4 were found more frequently in pT1 ADCs (Figure 4), and showed a borderline difference in OCT4 expression (p < 0.05, p = 0.0429). In 90 SqCCs, 25 cases were pT1, 28 cases were pT2, and 37 cases were pT3/4 tumors. The expressions of SALL4 and OCT4 showed different patterns (Figure 5). The stronger SALL4 expression was found in all different stages of SqCCs. But, this pattern was not found in OCT4 expressions. The stronger OCT4 expression was more frequently detected in pT3/4 SqCCs. However, the statistical analysis did not found significant differences between groups.

We also correlated expression of SALL4 and OCT4 with patients' survival time (Table 6). Although patients with tumors that expressed SALL4 and OCT4 seemed to have short survival time, we did not find significant differences of survival time among different groups of patients.

**Table 6:** **Correlation of SALL4 and OCT4 expression with patients' survival time**

Markers		Adenocarcinoma		Squamous cell carcinomas	
		(n = 77)		(n = 90)	
		Average survival months(cases)	P value	Average survival months(cases)	P value
SALL4	Negative	35.63 ± 3.18 (n = 51)	0.9866	36.98 ± 4.71 (n = 56)	0.3684
	Positive	35.50 ± 6.85 (n = 26)		29.91 ± 6.23 (n = 34)	
OCT4	Negative	38.73 ± 4.24 (n = 47)	0.1666	35.75 ± 4.54 (n = 67)	0.4815
	Positive	30.38 ± 4.21 (n = 29)		30.13 ± 6.48 (n = 23)	

### The potential relationship of SALL4 and OCT4 in primary NSCLC

In our study, the expression of SALL4 and OCT4 is different in different subtypes of NSCLC. In primary ADCs, the stronger expression of SALL4 and OCT4 in tumor tissue was 7.8% and 9.1%. In primary SqCCs, the stronger expressions of SALL4 and OCT4 were 16.7% and 0%, respectively. The stronger expression of SALL4 is more frequently detected in SqCCs than in ADCs, whereas, the stronger expression of OCT4 is more frequent detected in lung ADCs. The Fisher's exact test showed that the stronger expression of SALL4 (with score 2+) in SqCC and the stronger expression of OCT4 (with score 2+) in ADC were significantly different (p < 0.001, p = 0.0014). The stronger expression of SALL4 was inversely correlated with tumor differentiations in both ADC and SqCC. However, the expression of SALL4 was correlated with tumor stage and the expression of OCT4 only in SqCC. We also correlated the expression of SALL4 and OCT4 with the *EGFR* and *KRAS* mutations in ADCs. Among 77 ADCs, we only found 3 tumors had *EGFR* mutations and 5 tumors with *KRAS* mutations. Thus, we are unable to draw a conclusion based on this small number. Taken together, our findings suggest that different stem cell markers may be expressed and/or play different roles in different stages of NSCLCs.

An early study of SALL4 signaling pathway has found that SALL4 and OCT4 form a positive feedback loop [18]. In the network, SALL4 not only activates OCT4 expression, but is also positively regulated by OCT4 expression [18]. In addition to this positive regulatory mechanism, Yang et al. have also found that SALL4 is regulated by the auto-regulation mechanism of self-repression, which acts as a “gate keeper” or a “break” mechanism for the SALL4/OCT4 positive feedback loop [18]. Although both OCT4 and SALL4 have been considered to play important roles in oncogenesis, the differential expression of SALL4 and OCT4 in lung primary ADCs and SqCCs suggests that NSCLCs may be regulated differently in the SALL4 signaling pathway. Finally, similar to the study of breast cancer [24], we do not find that the expression of SALL4 is correlated with tumor stage or patients' survival time. The potential role of SALL4 and OCT4 in NSCLC needs to be further studied.

In a recent mechanism study of Kobayashi et al., they analyzed SALL4 mRNA levels in lung cancer tissue and found that SALL4 mRNA was highly expressed in lung cancers [33]. They also compared SALL4 mRNA levels in tumor tissue with non-neoplastic tissue from the same patient and found that 93% of tumor cases had a greater than two-fold increase in SALL4 mRNA. In the study, they also found that *SALL4* siRNA treatment in lung cancer cells dramatically inhibited cell growth via cell cycle arrest at the G1 and early S phases, indicating that SALL4 might be involved in the G1 phase transition of the cell cycle. These studies suggest that *SALL4* expression may play a potential role in the regulation of lung cancer development.

## Conclusions

We examined the expression of SALL4 and OCT4 in NSCLC tumor tissue using TMA and IHC stains. We found that both SALL4 and OCT4 were expressed in a subset of primary NSCLC, including both ADCs and SqCCs. The stronger expression of SALL4 is more frequently detected in SqCCs than in ADCs. In contrast, the stronger expression of OCT4 is more frequent detected in lung ADCs. In primary ADCs, the stronger expression of SALL4 and OCT4 was 7.8% and 9.1%. The stronger expression of SALL4 was inversely correlated with tumor differentiation. In primary SqCCs, the stronger expressions of SALL4 and OCT4 were 16.7% and 0%, respectively. The expression of SALL4 correlated with the expression of OCT4, but inversely correlated with the tumor stage in SqCCs. Taken together, our findings suggest that different stem cell markers may be expressed and/or play different roles in different subtypes of NSCLCs. In addition, our data also suggests that NSCLC should be added to the differential diagnosis of SALL4/OCT4 positive neoplasms, particularly in patients with mediastinal tumors. Future studies are necessary to clarify the potential role of SALL4 and OCT4 in NSCLCs.

## Authors’ original submitted files for images

Below are the links to the authors’ original submitted files for images. Authors’ original file for figure 1 Authors’ original file for figure 2 Authors’ original file for figure 3 Authors’ original file for figure 4 Authors’ original file for figure 5

## References

[1] Siegel R, Ma J, Zhou Z, Jemal A (2014). Cancer statistics, 2014. CA Cancer J Clin.

[2] Travis WD, Brambilla E, Muller-Hermelink HK, CC H (2004). Pathology and Genetic of Tumors of the Lung, Pleura, Thymus and Heart.

[3] Travis WD, Brambilla E, Noguchi M, Nicholson AG, Geisinger KR, Yatabe Y, Beer DG, Powell CA, Riely GJ, Van Schil PE, Garg K, Austin JH, Asamura H, Rusch VW, Hirsch FR, Scagliotti G, Mitsudomi T, Huber RM, Ishikawa Y, Jett J, Sanchez-Cespedes M, Sculier JP, Takahashi T, Tsuboi M, Vansteenkiste J, Wistuba I, Yang PC, Aberle D, Brambilla C, Flieder D (2011). International Association for the Study of Lung Cancer/American Thoracic Society/European Respiratory Society international multidisciplinary classification of lung adenocarcinoma. J Thorac Oncol.

[4] Chen HY, Yu SL, Chen CH, Chang GC, Chen CY, Yuan A, Cheng CL, Wang CH, Terng HJ, Kao SF, Chan WK, Li HN, Liu CC, Singh S, Chen WJ, Chen JJW, Yang PC (2007). A five-gene signature and clinical outcome in non-small-cell lung cancer. N Engl J Med.

[5] Paez JG, Jänne PA, Lee JC, Tracy S, Greulich H, Gabriel S, Herman P, Kaye FJ, Lindeman N, Boggon TJ, Naoki K, Sasaki H, Fujii Y, Eck MJ, Sellers WR, Johnson BE, Meyerson M (2004). EGFR mutations in lung cancer: correlation with clinical response to gefitinib therapy. Science.

[6] Coate LE, John T, Tsao MS, Shepherd FA (2009). Molecular predictive and prognostic markers in non-small-cell lung cancer. Lancet Oncol.

[7] Soda M, Choi YL, Enomoto M, Takada S, Yamashita Y, Ishikawa S, Fujiwara S, Watanabe H, Kurashina K, Hatanaka H, Bando M, Ohno S, Ishikawa Y, Aburatani H, Niki T, Sohara Y, Sugiyama Y, Mano H (2007). Identification of the transforming EML4-ALK fusion gene in non-small-cell lung cancer. Nature.

[8] Li QK, Singh A, Biswal S, Askin F, Gabrielson E (2011). *KEAP1* gene mutations and NRF2 activation are common in pulmonary papillary adenocarcinoma. J Hum Genet.

[9] Li QK, Gabrielson E, Zhang H (2012). Application of glycoproteomics for the discovery of biomarkers in lung cancer. Proteomics: Clinical App.

[10] Munfus-McCray D, Cui M, Zhang Z, Askin F, Gabrielson E, Li QK (2013). Comparison of *EGFR* and *KRAS* mutations in primary and unpaired metastatic lung adenocarcinoma with potential chemotherapy effect. Human Pathol.

[11] Li QK, Gabrielson E, Askin F, Chan DW, Zhang H (2013). Glycoproteomics using fluid based specimens in the discovery of lung cancer protein biomarkers. Promise and challenge. Proteomics Clinical App.

[12] Eramo A, Haas TL, De Maria R (2010). Lung cancer stem cells: tools and targets to fight lung cancer. Oncogene.

[13] Wang JC, Dick JE (2005). Cancer stem cells: lessons from leukemia. Trends Cell Biol.

[14] Klonisch T, Wiechec E, Hombach-Klonisch S, Ande SR, Wesselborg S, Schulze-Osthoff K, Los M (2008). Cancer stem cell markers in common cancers - therapeutic implications. Trends Mol Med.

[15] Kitamura H, Okudela K, Yazawa T, Sato H, Shimoyamada H (2009). Cancer stem cell: implications in cancer biology and therapy with special reference to lung cancer. Lung Cancer.

[16] Zhou Q, Chipperfield H, Melton DA, Wong WH (2007). A gene regulatory network in mouse embryonic stem cells. Proc Natl Acad Sci U S A.

[17] Zhang J, Tam WL, Tong GQ, Wu Q, Chan HY, Soh BS, Lou Y, Yang J, Ma Y, Chai L, Ng HH, Lufkin T, Robson P, Lim B (2006). Sall4 modulates embryonic stem cell pluripotency and early embryonic development by the transcriptional regulation of Pou5f1. Nat Cell Biol.

[18] Yang J, Gao C, Chai L, Ma Y (2010). A novel SALL4/OCT4 transcriptional feedback network for pluripotency of embryonic stem cells. PLoS One.

[19] Cui W, Kong NR, Ma Y, Amin HM, Lai R, Chai L (2006). Differential expression of the novel oncogene, SALL4, in lymphoma, plasma cell myeloma, and acute lymphoblastic leukemia. Mod Pathol.

[20] Deisch J, Raisanen J, Rakheja D (2011). Immunohistochemical expression of embryonic stem cell markers in malignant rhabdoid tumors. Ped Dev Pathol.

[21] Cao D, Humphrey PA, Allan RW (2009). SALL4 is a novel sensitive and specific marker for metastatic germ cell tumors, with particular utility in detection of metastatic yolk sac tumors. Cancer.

[22] Ikeda H, Sato Y, Yoneda N, Harada K, Sasaki M, Kitamura S, Sudo Y, Ooi A, Nakanuma Y (2012). α-Fetoprotein-producing gastric carcinoma and combined hepatocellular and cholangiocarcinoma show similar morphology but different histogenesis with respect to SALL4 expression. Hum Pathol.

[23] Forghanifard MM, Moghbeli M, Raeisossadati R, Tavassoli A, Mallak AJ, Boroumand-Noughabi S, Abbaszadegan MR (2013). Role of SALL4 in the progression and metastasis of colorectal cancer. J Biomed Sci.

[24] Kobayashi D, Kuribayshi K, Tanaka M, Watanabe N (2011). SALL4 is essential for cancer cell proliferation and is overexpressed at early clinical stages in breast cancer. Int J Oncol.

[25] Chiou SH, Wang ML, Chou YT, Chen CJ, Hong CF, Hsieh WJ, Chang HT, Chen YS, Lin TW, Hsu HS, Wu CW (2010). Coexpression of Oct4 and Nanog enhances malignancy in lung adenocarcinoma by inducing cancer stem cell-like properties and epithelial-mesenchymal transdifferentiation. Cancer Res.

[26] Edge SB, Byrd DR, Compton CC, Fritz AG, Greene FL, Trotti A (2010). AJCC Cancer Staging Manual.

[27] Kohlhase J, Schuh R, Dowe G, Kuhnlein RP, Jackle H, Schroeder B, Schulz-Schaeffer W, Kretzschmar HA, Kohler A, Muller U, Raab-Vetter M, Burkhardt E, Engel W, Stick R (1996). Isolation, characterization, and organ-specific expression of two novel human zinc finger genes related to the Drosophila gene spalt. Genomics.

[28] Kohlhase J, Schubert L, Liebers M, Rauch A, Becker K, Mohammed SN, Newbury-Ecob R, Reardon W (2003). Mutations at the SALL4 locus on chromosome 20 result in a range of clinically overlapping phenotypes, including Okihiro syndrome, Holt-Oram syndrome, acro-renal-ocular syndrome, and patients previously reported to represent thalidomide embryopathy. J Med Genet.

[29] Al-Baradie R, Yamada K, St Hilaire C, Chan WM, Andrews C, McIntosh N, Nakano M, Martonyi EJ, Raymond WR, Okumura S, Okihiro MM, Engle EC (2002). Duane radial ray syndrome (Okihiro syndrome) maps to 20q13 and results from mutations in SALL4, a new member of the SAL family. Am J Hum Genet.

[30] Ma Y, Cui W, Yang J, Qu J, Di C, Amin HM, Lai R, Ritz J, Krause DS, Chai L (2006). SALL4, a novel oncogene, is constitutively expressed in human acute myeloid leukemia (AML) and induces AML in transgenic mice. Blood.

[31] Deisch J, Raisanen J, Rakheja D (2011). Immunoexpression of SALL4 in Wilms tumors and developing kidney. Pathol Oncol Res.

[32] Bernhardt M, Galach M, Novak D, Utikal J (2012). Mediators of induced pluripotency and their role in cancer cells - current scientific knowledge and future perspectives. Biotechnol J.

[33] Kobayashi D, Kuribayashi K, Tanaka M, Watanabe N (2011). Overexpression of SALL4 in lung cancer and its importance in cell proliferation. Oncol Rep.

