# Supplementary figures and images for: Expression of transcript factors SALL4 and OCT4 in a subset of non-small cell lung carcinomas (NSCLC)
Source: Transl Respir Med. 2014 Oct 2;2:10. doi: 10.1186/s40247-014-0010-7 (PMC4201749; doi:10.1186/s40247-014-0010-7)

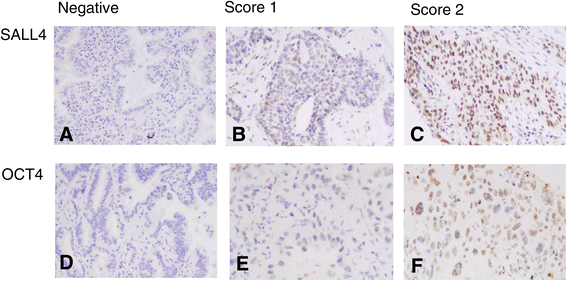

Supplement: Supplementary file 1 — Authors’ original file for figure 1 [file 40247_2014_10_MOESM1_ESM.gif]

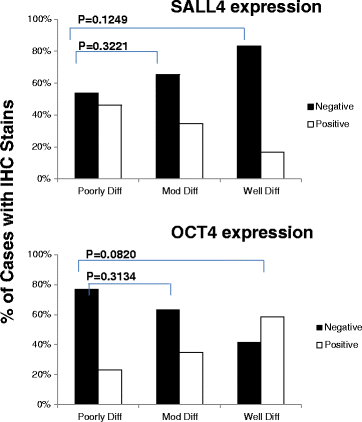

Supplement: Supplementary file 2 — Authors’ original file for figure 2 [file 40247_2014_10_MOESM2_ESM.gif]

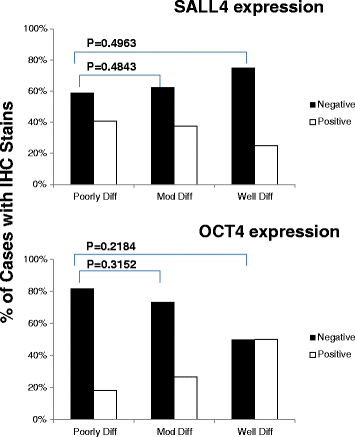

Supplement: Supplementary file 3 — Authors’ original file for figure 3 [file 40247_2014_10_MOESM3_ESM.gif]

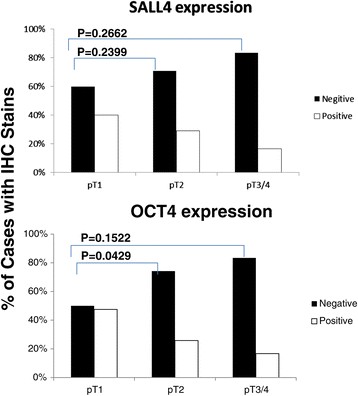

Supplement: Supplementary file 4 — Authors’ original file for figure 4 [file 40247_2014_10_MOESM4_ESM.gif]

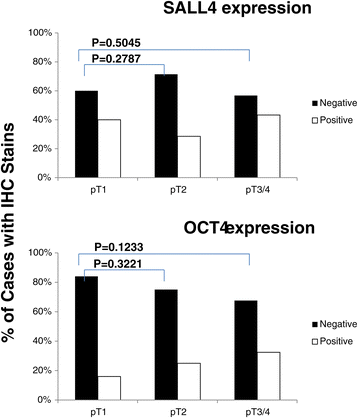

Supplement: Supplementary file 5 — Authors’ original file for figure 5 [file 40247_2014_10_MOESM5_ESM.gif]
